# Cross-cultural validity of the Pediatric Symptom Checklist – Youth Report (PSC-Y) in five ​Global ​South countries: Implications for equitable child mental health screening

**DOI:** 10.1186/s12916-026-05383-w

**Published:** 2026-11-04

**Authors:** Florian Scharpf, Milena Sievert, Katharina Mattonet, David Goretzko, Emiles Joseph, Amoah Kwaku Karikari, Alaptagin Khan, Anette Kirika, Charles Magoba Muwonge, Mabula Nkuba, Anke Hoeffler, Tobias Hecker

**Affiliations:** 1 Department of Psychology, Bielefeld University, P. O. Box 100131, 33501 Bielefeld, Germany; 2 Institute for Interdisciplinary Research on Conflict and Violence, Bielefeld University, Bielefeld, Germany; 3 Department of Psychology, Goethe University Frankfurt, Frankfurt, Germany; 4 P4H Global, Cap-Haitien, Haiti; 5 University of Energy and Natural Resources, Sunyani, Ghana; 6 Khyber Medical University, Peshawar, Pakistan; 7 Shifa International Hospitals, Islamabad, Pakistan; 8 McLean Hospital, Boston, USA; 9 Harvard Medical School, Boston, USA; 10 Department of Educational Foundations and Psychology, Mbarara University of Science and Technology, Mbarara, Uganda; 11 Faculty of Education, Uganda Martyrs University, Nkozi, Uganda; 12 Department of Educational Psychology and Curriculum Studies, University of Dar es Salaam (Dar es Salaam University College of Education), Dar es Salaam, Tanzania; 13 Department of Politics and Public Administration, University of Konstanz, Konstanz, Germany

**Keywords:** Measurement invariance, Pediatric Symptom Checklist (PSC-Y), Children and adolescents, Mental health, Emotional and behavioral problems, Global South, Cross-cultural validation, Screening, Gender

## Abstract

**Background:**

Despite a high global burden of mental health disorders in childhood and adolescence, access to adequate care remains limited, particularly in low- and middle-income countries​. Valid and reliable self-report instruments are essential for early identification and equitable care, yet measurement invariance as a prerequisite for valid group comparisons remains understudied. The Pediatric Symptom Checklist (PSC) is a brief, open-access screening tool with practical advantages for global use. This study examined the factor structure and measurement invariance of the 35-item PSC youth self-report (PSC-Y) across five Global South countries.

**Methods:**

We analyzed baseline data from 5,810 children and adolescents (age 6–18 years) participating in school-based randomized controlled trials in Ghana, Tanzania, Uganda, Haiti, and Pakistan. Using confirmatory factor analysis (CFA) and exploratory structural equation modeling (ESEM), we tested competing models (unidimensional, three-factor, second-order, bifactor). Multi-group CFA examined measurement invariance across countries. Within each of the five countries, we tested invariance across gender and across age groups in each of the three African countries where both primary and secondary students were represented, using school level as a proxy for developmental stage. Multiple Indicators Multiple Causes (MIMIC) models served as sensitivity analyses.

**Results:**

A strong general factor emerged across all countries (omega hierarchical > 0.90). Configural invariance was supported across countries, but metric invariance failed, with items related to empathy, rule-breaking, and anger expression showing differential functioning. Full metric, scalar, and strict invariance were achieved across gender in all five countries. The Tanzania and Uganda samples demonstrated full invariance across school level as a proxy for age, while Ghana required partial invariance after freeing five items (e.g., empathy, fighting). MIMIC models supported these findings.

**Conclusions:**

The PSC-Y exhibits a strong general factor across diverse Global South settings, supporting total score use for screening within countries. However, because metric invariance was not established across countries, cross-country comparisons of PSC-Y scores are not methodologically defensible. Within countries, full gender invariance validates direct comparisons between boys and girls. Findings underscore the need for ongoing local validation and cultural contextualization for advancing equitable global mental health research and practice.

**Supplementary Information:**

The online version contains supplementary material available at https://doi.org/10.1186/s12916-026-05383-w.

## Background

The mental health of children and adolescents is a cornerstone of their development and future wellbeing. The Convention on the Rights of the Child [1] emphasizes children’s fundamental right to good mental health, which encompasses access to quality care, supportive environments, and protection from harm. However, prevalence rates of mental health disorders in childhood and adolescence have been on the rise worldwide, accounting for a substantial share of the global burden of disease in these age groups [2–4]. Mental health problems in childhood and adolescence are consistently linked to adverse long-term outcomes in later life, including mental and physical health problems, interpersonal difficulties, and poor academic achievement [5–7]. Despite these known debilitating consequences and increasing recognition of child mental health as a global priority, a significant care gap persists, particularly in low- and middle-income countries (LMICs) where access to screening and support services remains limited [8]. Closing this gap requires efficient, scalable, and culturally valid instruments to enable early identification of children in need of support and to ensure equitable access to mental health care across diverse populations. Given that children can provide valid and reliable information on their mental health and wellbeing [9], self-report instruments are essential to this effort.

A fundamental consideration for global mental health research and practice is that Western-developed screening instruments may not fully capture local idioms of distress, and their uncritical application risks imposing external frameworks that may miss locally salient forms of suffering [10–12]. Yet validated, accessible tools are urgently needed in Global South settings, where up to 85% of those with mental health conditions receive no treatment [13]. While developing new instruments from the ground up is desirable, it may limit the international applicability of the measure and not always be feasible because it requires time, funding, and local expertise that are often in short supply [14]. Rigorous psychometric evaluation of the most useful available instruments, accompanied by careful translation and cultural adaptation, represents a possible compromise in the interim [12]. Rather than assuming universal validity, such efforts should treat cross-cultural validity as an empirical question to be tested, with the goal of informing appropriate use, identifying limitations, and highlighting sources of conceptual differences at the item level.

However, the cross-group application of mental health instruments introduces a fundamental methodological challenge. Children’s expression and reporting of psychological symptoms are deeply influenced by cultural norms, language, socio-emotional socialization, gender roles, and developmental stage [15, 16]. For instance, internalizing distress may manifest somatically in some cultures [17], and interpretations of externalizing behaviors vary with local parenting practices and cultural norms [18]. Moreover, externalizing behaviors show consistent gender differences, with boys exhibiting higher rates than girls throughout childhood and adolescence [19], and symptoms may present differently across development, with younger children expressing distress through behavior whereas adolescents may report more internalizing symptoms [20]. Consequently, observed score differences between groups—whether defined by country, culture, gender, or age—could reflect genuine variation in the underlying construct. However, they could also arise from systematic measurement bias if the instrument does not function equivalently across groups. For instance, findings of a systematic review suggest that items of child and adolescent depression scales may perform differently across gender and age groups [21]. To distinguish between these possibilities and ensure valid cross-group comparisons, researchers must establish measurement invariance—the statistical property that an instrument measures the same construct with the same underlying structure and item functioning across different populations [22]. Invariance testing proceeds hierarchically, from configural (same factor structure) to metric (equivalent factor loadings, allowing correlation comparisons) to scalar (equivalent item intercepts, necessary for mean comparisons). Without demonstrated scalar invariance, comparing scores across groups is scientifically untenable [23], and clinical decisions or resource allocation based on such comparisons risk being misguided or inequitable.

The Strengths and Difficulties Questionnaire (SDQ) and the Youth Self-Report (YSR) are the most commonly used and evaluated self-report instruments for screening mental health problems in global contexts [24–27]. However, a systematic review highlighted a significant lack of evidence on cross-cultural measurement invariance for most available self-report scales for child and adolescent psychopathology [28]. The difficulty of achieving cross-cultural invariance is particularly salient for the SDQ. Studies consistently report limited invariance across countries and cultural contexts, with findings suggesting that observed score differences may reflect cultural biases rather than true variation in mental health status [29–31]. Several studies have also observed problems with replicating the proposed five-factor structure using the original items, recommending caution when using the SDQ as a screening tool [26, 32, 33].

Researchers have attributed these challenges partly to the SDQ’s inclusion of reverse-worded items, which often compromise factorial stability, as well as culturally dependent interpretations of key constructs [34]. An additional practical limitation is that the SDQ is not freely available for digital applications, restricting its accessibility for large-scale screening, particularly in lower-resource contexts. The YSR fares somewhat better in terms of invariance and other psychometric properties [28, 35], but its considerable length and the fact that it is not freely available similarly limit its utility for broad implementation. These findings underscore the need for alternative instruments with greater cross-cultural robustness and accessibility, and highlight the importance of rigorous invariance testing before deploying self-report tools in diverse global settings.

The Pediatric Symptom Checklist (PSC) [36] offers several advantages that make it a promising candidate for global mental health screening. It is open-access, has been translated into numerous languages, and avoids methodologically problematic reverse-worded items—addressing two key limitations identified in the SDQ. Available in 35-item (PSC-35) and 17-item (PSC-17) versions for parent-report and youth self-report (PSC-Y), the PSC is much shorter than comprehensive tools like the YSR or the Child Behavior Checklist [37].

The PSC conceptualizes psychological distress across internalizing, externalizing, and attention problems, and while originally developed as a unidimensional tool, a three-factor structure has been validated, primarily for the parent-report PSC-17 in Western settings [38, 39]. The short and long versions of the PSC-Y have been used in observational and intervention studies across diverse cultural contexts, including China [40], Botswana [41], Zambia [42], Lebanon [43], and Pakistan [44, 45]. Its brevity, accessibility, and straightforward item phrasing make it particularly suitable for large-scale international applications, especially in low-resource and multilingual contexts.

Despite its potential, existing psychometric research on the PSC reveals three critical limitations. First, focus has predominantly been on the PSC-17 (both parent- and youth-reported), with the 35-item PSC-Y receiving less attention. While the brevity of the 17-item version offers practical advantages for screening, the 35-item version may provide a more comprehensive assessment of psychological distress. Second, validation studies have largely been conducted in high-income, Western contexts [39, 46–48]. The few studies in non-Western settings, such as a study of the self-reported PSC-17 in Zambia [42], suggest alternative models (e.g., bifactor models) may provide better fit, highlighting the potential for cultural variation in the instrument’s structure. Third, and most critically, comprehensive tests of the PSC’s measurement invariance across culturally and socioeconomically diverse countries are absent from the literature to the best of our knowledge. This gap severely limits the PSC’s scientific utility for global mental health research and surveillance.

The present study directly addresses these gaps by examining the factor structure and measurement invariance of the 35-item PSC-Y in samples from five LMICs in the Global South: Ghana, Tanzania, Uganda, Haiti, and Pakistan. These countries represent diverse geographic regions (West Africa, East Africa, South Asia, and the Caribbean), cultural backgrounds, religious traditions, and socioeconomic contexts, yet they share the common challenges of high prevalence rates of child and adolescent mental health problems among children and adolescents (e.g., over 40% in East Africa [49]; 25% in Pakistan [44]) and severely limited resources for mental health care [50, 51]. We aimed to (1) identify the optimal latent factor structure within each national sample; and (2) test for measurement invariance across countries and, within countries, across gender and age groups. These comparisons are essential for ensuring equitable assessment across demographic subgroups and developmental stages. Based on prior research on the 17-item PSC-Y [42, 46, 47] and the conceptually similar SDQ [26], we hypothesized that a multidimensional model will provide the best fit in each country, and that invariance will be greater across demographic subgroups within countries than across the culturally distinct national samples. By establishing the level of measurement equivalence, this study will clarify whether and how the 35-item PSC-Y can be used for valid comparisons across and within countries, thereby contributing to the development of more equitable and psychometrically sound assessment tools for global child mental health.

## Methods

### Study design and data source

This study constitutes a secondary analysis of pooled baseline data from three independent cluster-randomized controlled trials conducted in Ghana, Tanzania, Uganda, Haiti, and Pakistan. The trials evaluated a school-based intervention aiming to reduce teachers’ use of violence against children and adolescents, and are registered with ClinicalTrials.gov (Ghana, Tanzania, Uganda: NCT04948580; Haiti: NCT05081622; Pakistan: NCT06001554). This study’s analyses were preregistered on OSF [https://osf.io/tvp8d/overview] prior to analyses. All three trials employed the 35-item PSC-Y, administered in a structured interview format, to assess child and adolescent mental health, enabling the construction of a pooled cross-cultural dataset. Detailed descriptions of the original study designs and data collection procedures have been published elsewhere [52–54].

### Sampling and procedures

The total pooled sample comprised 5,810 children and adolescents between the ages of 6 and 18 years, recruited from public primary and secondary schools across the five participating countries. The country-specific sample sizes were 1,167 participants from Ghana, 1,086 from Tanzania, 1,240 from Uganda, 1,257 from Haiti, and 1,060 from Pakistan. As shown in Table 1, the gender distribution was approximately balanced across all sites, with the proportion of female participants ranging from 49.4% in Haiti to 52.9% in Tanzania.

**Table 1:** Demographic characteristics by country

	Tanzania	Ghana	Uganda	Pakistan	Haiti
Sex, *n* (%)					
Male	512 (47.1)	576 (49.4)	586 (47.3)	521 (49.2)	636 (50.6)
Female	574 (52.9)	591 (50.6)	654 (52.7)	539 (50.8)	621 (49.4)
School type, *n (%)*					
Primary	555 (51.1)	560 (48.0)	628 (50.6)	–	1257 (100)
Secondary	531 (48.9)	607 (52.0)	612 (49.4)	1060 (100)	–
Age, *M (SD)*					
Overall	11.57 (2.99)	12.11 (2.48)	13.44 (2.77)	13.12 (1.27)	12.41 (2.03)
Primary	8.85 (1.13)	9.95 (1.46)	11.03 (1.37)	–	12.41 (2.03)
Secondary	14.40 (1.06)	14.10 (1.27)	15.91 (1.23)	13.12 (1.27)	–

In each country, a multi-stage sampling strategy was employed to ensure diversity in geographic, socioeconomic, and institutional characteristics. Schools were eligible for inclusion if they were public, had the required minimum number of students and staff for inclusion in the respective trial, and had not recently participated in similar training or violence prevention programs. Schools were mixed-gender in all countries except Pakistan, where single-gender schools were included to reflect the national educational context. Within selected schools, students from target grades were randomly selected from official class lists, with stratification by gender to ensure balanced representation. In Ghana, Tanzania, and Uganda, both primary and secondary school students were included, with 24 schools per country recruited and at least 40 students per school drawn from either the third year of primary school (third year of formal schooling) or the first year of secondary (Tanzania and Uganda; eighth year of formal schooling) or junior high school (Ghana; seventh year of formal schooling). In Haiti, all participants attended primary schools, with 36 schools selected and at least 28 students per school from grade four (fourth year of formal schooling). In Pakistan, only secondary schools participated, with 24 single-gender schools comprising 12 girls’ schools and 12 boys’ schools selected and at least 30 students per school from grade six (sixth year of formal schooling). These differences in school type were reflected in participant ages: as detailed in Table 1, primary school students were generally younger, with mean ages ranging from 8.85 to 12.41 years (SD = 1.13 to 2.03) across countries, while secondary school students had mean ages ranging from 13.12 to 15.90 years (SD = 1.06 to 1.27).

All structured interviews were conducted using standardized computer-assisted personal interviewing procedures by trained assessors. The assessment battery covered multiple domains of children’s lives, including mental health, well-being, interpersonal relationships, psychological competences, and exposure to violence at school and at home. Interviews were carried out in the language best understood by each participant to ensure comprehension and accurate responding: primarily Kiswahili in Tanzania; English or local languages such as Twi and Luganda in Ghana and Uganda, respectively; Kreyol in Haiti; and Urdu in Pakistan (see Additional file 1: Table S1 for a summary and the Open Science Framework (OSF) repository for complete translated versions: https://osf.io/tvp8d/overview). All measures, including the PSC-Y, were translated into the respective local languages using a blind forward and backward translation procedure. Ethical approval was obtained from all relevant institutional review boards in each country, and informed consent was collected from participants and their primary caregivers.

### Measures

Demographic information included participants’ self-reported age, assessed with an open-ended question, and gender measured with a binary item offering the response options female and male.

Emotional and behavioural problems were assessed using the 35-item PSC-Y, which was developed to identify psychological distress in children and adolescents and has been used with children and adolescents ages 9 and above [55]. It consists of 35 items describing behavioral or emotional symptoms that children may experience in everyday life (see Additional file 2: Table S2 for complete item wordings and their corresponding subscales). Each item is rated on a three-point Likert scale, with 0 indicating never, 1 indicating sometimes, and 2 indicating often. Respondents were asked to refer to the past month when answering the items. Item scores are summed to obtain a total score ranging from 0 to 70, with higher values indicating greater psychological distress. A commonly used cut-off for elevated risk of clinically significant mental health problems is a total score of 28 or above [44, 55].

The PSC-Y has demonstrated good psychometric properties in a range of cultural settings. In a community sample in Botswana, it showed high internal consistency with a Cronbach’s alpha of 0.86 and demonstrated adequate sensitivity and specificity in identifying children with emotional and behavioural problems [41]. In Pakistan, the Urdu version of the PSC-Y has been validated for use in public schools in the Rawalpindi district, showing high internal consistency (Cronbach’s α = 0.85) as well as good reliability and concurrent validity when compared against the SDQ [44].

To enhance the clarity and developmental appropriateness of the instrument in the study contexts of the original trials, two items were rephrased and clarified based on findings from pilot testing. Specifically, item 7, which originally reads “I act as if driven by a motor,” was rephrased to “I feel like I always have to move around and do something.” Item 19, originally “I am down on myself,” was revised to “I think badly of myself.” These adaptations followed best practices for culturally sensitive instrument development [56] and were designed to preserve construct equivalence while improving semantic clarity for young respondents across diverse settings.

### Data analyses

All statistical analyses were conducted using R [57] and RStudio [58]. The complete R syntax for all models is available on OSF [https://osf.io/tvp8d/overview]. The analysis proceeded in three stages: (1) establishing the factor structure of the PSC-Y within each country, (2) testing measurement invariance across countries, and (3) examining within-country invariance across gender and age subgroups. No missing data were present, so no imputation or deletion was necessary. Preliminary descriptive statistics were computed for all items, including means, standard deviations, skewness, kurtosis, and indicators of floor/ceiling effects. Within Ghana, Tanzania, and Uganda, age groups for invariance testing were defined based on school level (primary school or secondary/junior high school) as a proxy for developmental stage [42]. This approach is common in school-based developmental research [9, 59], where grade level reflects both chronological age and cumulative educational exposure. In Haiti and Pakistan, only one school level was represented (primary in Haiti, secondary in Pakistan) and there was therefore limited variation in age, precluding age group comparisons in these countries.

To identify the optimal factor structure, we conducted confirmatory factor analyses (CFA) and exploratory structural equation models (ESEM) separately for each country using the weighted least square mean and variance adjusted​ (WLSMV) estimator to account for ordinal data. Based on previous psychometric work on the PSC [38, 39, 42, 48, 60, 61], we tested several competing models: (a) a unidimensional model representing general psychological distress as a single latent construct; (b) a correlated three-factor model (internalizing, externalizing, and attention problems); (c) a second-order model with the three first-order factors loading on a general distress factor; and (d) a bifactor model with a general factor and three orthogonal specific factors. Following DiStefano et al. [60], we initially specified a second-order model with cross-loadings for items 8 (“Daydream too much”) and 29 (“Do not listen to rules”). However, as this model failed to converge in Pakistan and produced negative latent variances with low factor variance in the other countries, a second-order model without cross-loadings was also tested. Bifactor models required a fixed orthogonal specification to achieve convergence in four of five countries, though these models produced negative observed variances and were interpreted with caution. ESEM models included (d) a target-rotated three-factor solution and (e) a bifactor ESEM solution [62, 63]. These models allow items to load on multiple factors, providing a less restrictive alternative to CFA.

Model fit was assessed using multiple global fit indices, with acceptable fit defined as comparative fit index (CFI) values ≥ 0.90, root mean square error of approximation (RMSEA) values ≤ 0.08, and standardized root mean square residual (SRMR) < 0.08, and good fit as CFI ≥ 0.95 and RMSEA values ≤ 0.05, in line with conventional standards [64–66]. While such cutoffs have been criticized repeatedly for being misleading in some data conditions [67], they remain widely used as a pragmatic rule-of-thumb for an approximate model fit evaluation. Competing models were compared using chi-square difference tests and changes in fit indices, with CFI > 0.01 and RMSEA > 0.015 indicating worse fit [68]. Local fit was assessed by inspecting standardized residuals and factor loadings, with standardized residuals > |4.0| or factor loadings < 0.30 indicating potential local misfit [69, 70]. For ESEM solutions, we evaluated primary loadings (> 0.40), cross-loadings, and theoretical interpretability [60, 63]. The best-fitting model in each country was retained for invariance analyses. Internal consistency was assessed using Cronbach’s alpha. For bifactor models, model-based omega coefficients were computed to assess the reliability of the general factor and the specific factors, as recommended for multidimensional structures where traditional alpha may be misleading [71].

Measurement invariance across countries was tested hierarchically using the best-fitting common model: configural invariance (same factor structure), metric invariance (equal factor loadings), scalar invariance (equal item intercepts), and strict invariance (equal residual variances) [22, 23]. The respective invariance levels were supported if ΔCFI ≤ 0.01 and ΔRMSEA ≤ 0.015 [68]. Modification indices were used to explore partial invariance when full invariance was not supported. Within countries, the same hierarchical procedure was applied to test invariance across gender (in all five countries) and age subgroups (in the three African countries).

Multiple Indicators Multiple Causes (MIMIC) models were estimated as sensitivity analyses to complement the primary invariance analyses and provide additional evidence for the robustness of the findings. This approach offers an alternative method for detecting differential item functioning (DIF) by modeling differences across countries, gender and age subgroups as direct effects on item responses [72].

## Results

### Descriptives

Descriptive statistics for the PSC-Y total scores are presented in Table 2. Across all countries, mean total scores ranged from 13.2 in Ghana and Pakistan to 16.8 in Haiti, with standard deviations ranging from 7.98 to 8.66. The minimum score of zero was observed in all countries, while maximum scores varied from 43 in Ghana to 62 in Haiti. Using the cut-off of 28 or above, the proportion of children scoring above the threshold ranged from 5.9% in Pakistan to 10.4% in Haiti.

**Table 2:** Descriptive statistics of PSC-35 total scores by country

Country	*n*	M	SD	Min	Max	% ≥ 28
Tanzania	1086	13.8	8.40	0	48	6.9
Ghana	1167	13.2	8.66	0	43	8.2
Uganda	1240	15.7	8.49	0	54	9.7
Pakistan	1060	13.2	7.98	0	46	5.9
Haiti	1257	16.8	8.49	0	62	10.4

Item-level descriptive statistics, including floor and ceiling effects, skewness, and kurtosis, are reported in full in Additional file 3: Tables S3a and S3b. Across countries, item means indicated generally low to moderate levels of symptom endorsement, with sufficient variability to support subsequent modeling. Floor effects were most pronounced for internalizing items, particularly in Pakistan, Haiti, and Uganda, where more than 80% of respondents selected the lowest response category for several items. Ceiling effects were minimal overall, with only item 23 (“want to be with parents more than before”) showing comparatively higher endorsement rates, most notably in Pakistan and Haiti. Skewness and kurtosis values were largely within acceptable limits for ordinal data, supporting the use of the WLSMV estimator in subsequent analyses. Items with pronounced floor effects displayed elevated positive skew and kurtosis, as expected given their distributions. Between-country comparisons revealed broadly similar response patterns overall, though externalizing items showed slightly higher means in Tanzania and Uganda, while attention-related items tended to be endorsed somewhat more frequently in Pakistan and Haiti. Overall, the descriptive analyses indicate that the 35 PSC-Y items exhibit adequate distributional properties for categorical modeling, with limited ceiling effects, interpretable skewness and kurtosis, and sufficient variability across diverse cultural settings.

### Confirmatory factor analyses (CFA) and exploratory structural equation models (ESEM)

CFA were conducted separately for each country to examine the latent structure of the PSC-35. Table 3 summarizes global fit indices for the unidimensional, three-factor, second-order (with and without cross-loadings), and bifactor models. Across countries, the three-factor and second-order models consistently demonstrated relatively good fit, whereas the unidimensional model generally performed less well. Bifactor models in Tanzania, Ghana, Uganda, and Haiti required a fixed orthogonal specification to achieve convergence but produced negative observed variances; in Pakistan, the bifactor model converged without issue.

**Table 3:** Model fit indices for PSC-35 within countries

Model	χ²	df	CFI	TLI	RMSEA	SRMR
Tanzania						
One-Factor	1738	560	0.947	0.943	0.044	0.075
Three-Factor	1649	557	0.950	0.947	0.042	0.073
Second-Order (cross)^a^	1636	553	0.951	0.947	0.042	0.073
Second-Order (simple)^a, b^	1649	557	0.950	0.947	0.042	0.073
Bifactor (fixed)^c^	1980	529	0.934	0.926	0.050	0.080
Ghana						
One-Factor	2197	560	0.955	0.952	0.050	0.075
Three-Factor	1766	557	0.967	0.965	0.043	0.069
Second-Order (cross)^a^	1736	553	0.968	0.965	0.043	0.069
Second-Order (simple)^a, b^	1766	557	0.967	0.965	0.043	0.069
Bifactor (fixed)^c^	2902	529	0.935	0.927	0.062	0.088
Uganda						
One-Factor	1558	560	0.958	0.956	0.038	0.066
Three-Factor	1448	557	0.963	0.960	0.036	0.063
Second-Order (cross)^a^	1430	553	0.963	0.961	0.036	0.063
Second-Order (simple)^a, b^	1448	557	0.963	0.960	0.036	0.063
Bifactor (fixed)^c^	2211	529	0.930	0.921	0.051	0.076
Pakistan						
One-Factor	1561	560	0.945	0.942	0.041	0.082
Three-Factor	1375	557	0.955	0.952	0.037	0.076
Second-Order (cross)^a, d^	-	-	-	-	-	-
Second-Order (simple)^a, b^	1375	557	0.955	0.952	0.037	0.076
Bifactor (free)	1191	525	0.964	0.959	0.035	0.072
Haiti						
One-Factor	2883	560	0.891	0.884	0.058	0.088
Three-Factor	2698	557	0.899	0.893	0.055	0.087
Second-Order (cross)^a^	2673	553	0.900	0.893	0.055	0.087
Second-Order (simple)^a, b^	2698	557	0.899	0.893	0.055	0.087
Bifactor (fixed)^c^	2965	529	0.886	0.871	0.061	0.091

Note. χ² = chi-square; df = degrees of freedom; CFI = Comparative Fit Index; TLI = Tucker-Lewis Index; RMSEA = Root Mean Square Error of Approximation; SRMR = Standardized Root Mean Square Residual

^a^ The second-order model with cross-loadings (labeled “cross”) includes items 8 and 29 loading on all three first-order factors (Internalizing, Externalizing, and Attention), following DiStefano et al. (2017). The second-order model without cross-loadings (labeled “simple”) includes only the primary factor loadings

^b^ The second-order model without cross-loadings is statistically equivalent to the three-factor model, as indicated by identical fit statistics and degrees of freedom (df = 557). This reflects the mathematical equivalence when a second-order factor is just-identified with three first-order factors

^c^ Bifactor models in Tanzania, Ghana, Uganda, and Haiti used fixed orthogonal specification (variances fixed to 1) to achieve convergence, but each produced negative observed variances for three items. Pakistan’s bifactor model was freely estimated and showed no estimation problems

^d^ The second-order model with cross-loadings did not converge in Pakistan

As shown in Table 4, the second-order model without cross-loadings provided a stable and comparable structure across all countries, with comparably good fit in most settings (CFI range: 0.899-0.967). In this model, the three first-order factors (internalizing, externalizing, and attention) loaded strongly on the higher-order general factor across all countries, with standardized loadings ranging from 0.81 to 0.99 (see Additional file 4: Table S4). The correlated three-factor model, which is statistically equivalent to the second-order model, demonstrated identical fit indices and yielded high factor correlations across all samples (see Additional file 5: Table S5). Correlations ranged from 0.74 to 0.91, with mean correlations of 0.81 (Internalizing-Externalizing), 0.87 (Internalizing-Attention), and 0.83 (Externalizing-Attention), indicating substantial shared variance among the domains. The model demonstrated rather strong fit in Tanzania (CFI = 0.950, RMSEA = 0.042, SRMR = 0.073), Ghana (CFI = 0.967, RMSEA = 0.043, SRMR = 0.069), Uganda (CFI = 0.963, RMSEA = 0.036, SRMR = 0.063), and Pakistan (CFI = 0.955, RMSEA = 0.037, SRMR = 0.076). Fit was somewhat weaker but could still be considered acceptable in Haiti (CFI = 0.899, RMSEA = 0.055, SRMR = 0.087).

**Table 4:** CFA-model comparison

Model Comparison	ΔCFI	ΔRMSEA	Δχ²(df)	*p*-value
Tanzania				
1-Factor vs. 3-Factor	0.003	− 0.002	70.34 (3)	< 0.001***
3-Factor vs. Second-Order (Cross)	0.001	0	11.53 (4)	0.021*
3-Factor vs. Second-Order (Simple)^a^	0	0	< 0.01	-
Second-Order (Cross vs. Simple)	0.001	0	11.53 (4)	0.021*
3-Factor vs. Bifactor (Fixed)^b^	− 0.016	0.008	-	-
Ghana				
1-Factor vs. 3-Factor	0.012	− 0.007	217.65 (3)	< 0.001***
3-Factor vs. Second-Order (Cross)	0.001	0	22.40 (4)	< 0.001***
3-Factor vs. Second-Order (Simple)^a^	0	0	< 0.01	-
Second-Order (Cross vs. Simple)	0.001	0	22.40 (4)	< 0.001***
3-Factor vs. Bifactor (Fixed)^b^	− 0.032	0.019	-	-
Uganda				
1-Factor vs. 3-Factor	0.005	− 0.002	88.68 (3)	< 0.001***
3-Factor vs. Second-Order (Cross)	0	0	17.60 (4)	0.002**
3-Factor vs. Second-Order (Simple)^a^	0	0	< 0.01	-
Second-Order (Cross vs. Simple)	0	0	17.60 (4)	0.002**
3-Factor vs. Bifactor (Fixed)^b^	-0.33	0.015	-	-
Pakistan				
1-Factor vs. 3-Factor	0.010	− 0.004	110.61 (3)	< 0.001***
3-Factor vs. Second-Order (Cross)^c^	-	-	-	-
3-Factor vs. Second-Order (Simple)^a^	0	0	< 0.01	-
Second-Order (Cross vs. Simple)^c^	-	-	-	-
3-Factor vs. Bifactor (Free)	0.009	− 0.002	154.72 (32)	< 0.001***
Haiti				
1-Factor vs. 3-Factor	0.008	− 0.002	150.35 (3)	< 0.001***
3-Factor vs. Second-Order (Cross)	0.001	0	23.44 (4)	< 0.001***
3-Factor vs. Second-Order (Simple)^a^	0	0	< 0.01	-
Second-Order (Cross vs. Simple)	0.001	0	23.44	< 0.001***
3-Factor vs. Bifactor (Fixed)^b^	-0.13	0.006	-	-

Note: *p < .05, **p < .01, **p < .001

^a^ The second-order model without cross-loadings is statistically equivalent to the 3-factor model, as indicated by identical fit statistics and degrees of freedom (df = 557). The Δχ² values near zero with 0 degrees of freedom reflect this mathematical equivalence

^b^ Bifactor models in Tanzania, Ghana, Uganda, and Haiti used fixed orthogonal specification (variances fixed to 1) to achieve convergence, but each produced negative observed variances for 3 items. Pakistan’s bifactor model was freely estimated and showed no estimation problems

^c^ The second-order model with cross-loadings (labeled “Cross,” including items 8 and 29 on all factors following DiStefano et al., 2017) did not converge in Pakistan

In addition to these confirmatory models, ​ESEM models were estimated to evaluate more flexible factor solutions. Table 5 presents fit indices for target-rotated ESEM and bifactor ESEM models across all countries. Reliability estimates for the bifactor ESEM models are reported in Additional file 6: Table S6. The target-rotated ESEM models demonstrated appropriate fit in most countries, with CFI values ranging from 0.887 (Haiti) to 0.955 (Uganda) and RMSEA values from 0.042 (Uganda) to 0.063 (Haiti). The bifactor ESEM models showed substantially stronger and more consistent fit across all five countries, with CFI values ranging from 0.926 (Haiti) to 0.978 (Ghana) and RMSEA values from 0.032 (Uganda) to 0.049 (Haiti). Critically, the bifactor ESEM converged without issue in Pakistan (CFI = 0.964, RMSEA = 0.035, SRMR = 0.072), showing better model fit than the second-order models in that setting. Model-based McDonald’s omega coefficients (Additional file 6: Table S6) indicated that the general factor (G) accounted for the majority of reliable variance in the bifactor ESEM, with omega hierarchical values ranging from 0.907 in Haiti to 0.927 in Ghana. In contrast, the specific factors (Internalizing, Externalizing, Attention) contributed relatively little unique variance after controlling for the general factor, with omega hierarchical values for specific factors ranging from 0.003 to 0.699 across countries.

**Table 5:** Fit indices for ESEM models by country

Model	CFI	TLI	RMSEA	SRMR
Tanzania				
Target-rotated ESEM	0.943	0.930	0.049	0.075
Bifactor-ESEM	0.969	0.965	0.034	0.066
Ghana				
Target-rotated ESEM	0.953	0.942	0.055	0.075
Bifactor-ESEM	0.978	0.975	0.036	0.064
Uganda				
Target-rotated ESEM	0.955	0.945	0.042	0.066
Bifactor-ESEM	0.971	0.968	0.032	0.058
Pakistan				
Target-rotated ESEM	0.941	0.928	0.046	0.082
Bifactor-ESEM	0.964	0.959	0.035	0.072
Haiti				
Target-rotated ESEM	0.887	0.862	0.063	0.088
Bifactor-ESEM	0.926	0.917	0.049	0.077

Note. CFI = Comparative Fit Index; TLI = Tucker–Lewis Index; RMSEA = Root Mean Square Error of Approximation; SRMR = Standardized Root Mean Square Residual

### Model selection for invariance testing

Although the bifactor ESEM models demonstrated excellent fit within each country, they proved unsuitable for multi-group invariance testing. When we attempted to extend these models to cross-country comparisons, we encountered estimation problems including convergence failures in several subgroup analyses (e.g., gender comparisons within Pakistan) and negative variance estimates for multiple items, indicating model misspecification or empirical underidentification. These issues reflect the substantial complexity of bifactor ESEM models which estimate general factor loadings, specific factor loadings, and cross-loadings for all items, and their limited feasibility for multi-group applications where equality constraints are imposed across groups [73].

Given these estimation challenges, we selected the second-order CFA model without cross-loadings for cross-country invariance testing. This model represents a more parsimonious operationalization of the same underlying theoretical structure, a general factor with three subordinate dimensions (internalizing, externalizing, and attention problems), while enabling stable estimation across all groups. For within-country subgroup analyses (gender and age comparisons), we employed the three-factor correlated model. The second-order model proved problematic for these analyses due to convergence failures and negative variance estimates in smaller subgroups. The three-factor correlated model is statistically equivalent to the second-order model without cross-loadings (identical fit indices and degrees of freedom) and proved more robust in the smaller samples required for subgroup comparisons. The high correlations among the factors in the three-factor model reflect the same shared variance that the second-order model captures as a general factor.

### Measurement invariance across countries

Fit indices for the sequential invariance models for the second-order model without cross-loadings are presented in Table 6. The configural model, representing an unconstrained second-order structure, demonstrated good fit according to our pre-specified cutoffs (CFI = 0.950, RMSEA = 0.044, SRMR = 0.074), indicating that the overall factor configuration can be considered comparable across groups. When factor loadings were constrained to equality across all five countries (metric invariance), model fit declined substantially (ΔCFI = **–**0.041, ΔRMSEA = + 0.014), exceeding conventional cutoffs for non-invariance (Chen, 2007). This failure of metric invariance indicates that the strength of relationships between items and first-order factors differs significantly across countries.

**Table 6:** Measurement invariance of the PSC-35 across countries (simple second-order model)

Model	χ²	df	CFI	TLI	RMSEA	SRMR	ΔCFI	ΔRMSEA
Configural	8935	2785	0.950	0.946	0.044	0.074	—	—
Metric	14,019	2921	0.909	0.907	0.057	0.093	–0.041	+ 0.014
Scalar	10,193	2921	0.940	0.939	0.046	0.075	+​0.031	–0.011
Strict	15,508	3061	0.898	0.901	0.059	0.093	–0.042	+ 0.013

*Note. χ²* = Chi-square; *df* = degrees of freedom; CFI = Comparative Fit Index; TLI = Tucker–Lewis Index; RMSEA = Root Mean Square Error of Approximation; SRMR = Standardized Root Mean Square Residual. ΔCFI and ΔRMSEA represent fit changes relative to the preceding model. Metric invariance failed (ΔCFI = –0.041, exceeding the –0.01 cutoff), indicating that the PSC-Y does not measure the same construct with equivalent item-factor relationships across the five national samples. Consequently, scalar and strict invariance models are not interpretable. The scalar model’s anomalous improvement (ΔCFI = + 0.031) reflects model misspecification rather than evidence of invariance, as scalar invariance cannot be evaluated when metric invariance has not been established (Vandenberg & Lance, 2000). Only configural invariance is supported across countries

Inspection of modification indices revealed widespread non-invariance affecting multiple items across all factors and countries. Even after sequentially freeing the largest modification indices, model fit did not improve, suggesting that the non-invariance is systemic rather than isolated to a few problematic items. The pattern of high modification indices was distributed across all countries, with Tanzania (45), Ghana (31), and Uganda (17) showing the highest concentrations, and affected all first-order factors (Attention: 36, Externalizing: 35, Internalizing: 22). Items 31 (“Do not understand other people’s feelings”), 32 (“Tease others”), and 27 (“Have less fun with doing things”) emerged as consistently problematic across multiple countries.

Given that full metric invariance could not be established, and even partial metric invariance would require freeing an excessive number of parameters, only configural invariance is supported across the five countries. The scalar invariance model in Table 6 shows an apparent improvement in fit (ΔCFI = + 0.031), but as noted in the table this is not interpretable because scalar invariance cannot be evaluated when metric invariance has failed.

Post-hoc, we also examined measurement invariance across the three African countries (Tanzania, Ghana, and Uganda), which were drawn from the same trial and are culturally more similar than the full five-country set. Full results are presented in Additional file 7: Table S7. Metric invariance was not supported (ΔCFI = − 0.038, ΔRMSEA = + 0.015), mirroring the pattern observed in the full five-country comparison.

### Measurement invariance within countries by gender and school level

The PSC-35 demonstrated full measurement invariance across gender in all five countries. As shown in Table 7, configural models showed good fit in all samples, and all ΔCFI values across metric, scalar, and strict invariance tests were within the recommended − 0.01 cutoff. This indicates equivalent factor loadings, item thresholds, and residual variances for males and females across every national sample.

**Table 7:** Measurement invariance of the PSC-35 within countries by gender and school type (three-factor correlated model)

Grouping Variable	Model	χ²	df	CFI	TLI	RMSEA	SRMR	ΔCFI	ΔRMSEA
Tanzania									
Gender	Configural	2164.22	1114	0.954	0.951	0.042	0.086	—	—
	Metric	2333.88	1146	0.948	0.946	0.044	0.090	−0.006	+ 0.002
	Scalar	2287.85	1178	0.951	0.951	0.042	0.087	+ 0.003	−0.002
	Strict	2287.85	1178	0.951	0.951	0.042	0.087	0.000	0.000
School Type	Configural	2310.74	1114	0.945	0.941	0.045	0.092	—	—
	Metric	2488.76	1146	0.938	0.935	0.046	0.095	−0.007	+ 0.002
	Scalar	2566.62	1178	0.936	0.935	0.047	0.093	− 0.002	0.000
	Strict	2566.62	1178	0.936	0.935	0.047	0.093	0.000	0.000
Ghana									
Gender	Configural	2172.36	1114	0.972	0.970	0.040	0.078	—	—
	Metric	2341.52	1146	0.968	0.967	0.042	0.081	−0.004	+ 0.002
	Scalar	2301.11	1178	0.970	0.970	0.040	0.079	+ 0.002	−0.002
	Strict	2301.11	1178	0.970	0.970	0.040	0.029	0.000	0.000
School Type^a^	Configural	2476.70	1114	0.966	0.964	0.046	0.084	—	—
	Metric	2872.94	1141	0.957	0.956	0.051	0.090	− 0.009	+ 0.005
	Scalar	2812.27	1173	0.960	0.959	0.049	0.085	+ 0.002	− 0.002
	Strict	2812.27	1173	0.960	0.959	049	0.085	0.000	0.000
Uganda									
Gender	Configural	1912.86	1114	0.968	0.965	0.034	0.073	—	—
	Metric	2142.13	1146	0.960	0.958	0.037	0.077	−0.008	+ 0.003
	Scalar	2099.52	1178	0.963	0.962	0.036	0.074	+ 0.003	−0.002
	Strict	2099.52	1178	0.963	0.962	0.036	0.074	0.000	0.000
School Type	Configural	2010.88	1114	0.964	0.962	0.036	0.074	—	—
	Metric	2283.14	1146	0.955	0.953	0.040	0.079	−0.010	+ 0.004
	Scalar	2306.76	1178	0.955	0.955	0.039	0.076	0.000	−0.001
	Strict	2306.76	1178	0.955	0.955	0.039	0.076	0.000	0.000
Pakistan									
Gender	Configural	1868.15	1114	0.961	0.958	0.036	0.091	—	—
	Metric	2045.82	1146	0.954	0.952	0.039	0.096	− 0.008	+ 0.003
	Scalar	2077.70	1178	0.954	0.953	0.038	0.094	0.000	−0.001
	Strict	2077.70	1178	0.954	0.953	0.038	0.094	0.000	0.000
School Type^b^	—	—	—	—	—	—	—	—	—
Haiti									
Gender	Configural	3327.09	1114	0.901	0.894	0.056	0.097	—	—
	Metric	3538.14	1146	0.893	0.889	0.058	0.100	−0.008	+ 0.001
	Scalar	3541.97	1178	0.894	0.893	0.057	0.099	+ 0.001	−0.001
	Strict	3541.97	1178	0.894	0.893	0.057	0.099	0.000	0.000
School Type^b^	—	—	—	—	—	—	—	—	—

Note. χ² = Chi-square; df = degrees of freedom; CFI = Comparative Fit Index; TLI = Tucker–Lewis Index; RMSEA = Root Mean Square Error of Approximation; SRMR = Standardized Root Mean Square Residual. ΔCFI and ΔRMSEA represent fit changes relative to the preceding model. All models were estimated using the three-factor correlated model with WLSMV estimation and theta parameterization. Under this approach, residual variances are fixed to 1 for identification, rendering strict invariance (equal residuals) functionally equivalent to scalar invariance—hence the identical fit indices for scalar and strict models

^a^Ghana school type models include partial invariance with five items freed (items 31, 21, 10, 30 and 16)

^b^School type invariance was not examined in Pakistan and Haiti as each sample comprised a single school level (secondary in Pakistan, primary in Haiti)

Invariance across school level—serving as a proxy for developmental stage, with primary students representing middle childhood and secondary students representing adolescence—was examined in Tanzania, Ghana, and Uganda using the three-factor correlated model. As shown in Table 7, the pattern of age-related invariance varied across the three African samples. Tanzania demonstrated full invariance across all levels, with changes in fit indices well within conventional cutoffs (metric ΔCFI = -0.007, scalar ΔCFI = -0.002, strict ΔCFI = 0.000). Uganda met conventional cutoffs for full invariance, though the metric step was borderline (ΔCFI = -0.010), with scalar and strict invariance subsequently supported (ΔCFI = 0.000 for both). Ghana presented a more complex pattern. Full metric invariance was not supported (ΔCFI = -0.017), but partial metric invariance was achieved after freeing five items (items 31, 21, 10, 30 and 16) identified through modification indices. With these loadings freed, scalar invariance (ΔCFI = + 0.002) and strict invariance (ΔCFI = 0.000) were supported, indicating that aside from the freed parameters, the measurement properties appear to be equivalent across age groups in Ghana.

### MIMIC sensitivity analyses

A MIMIC model with the second-order PSC factor regressed on country dummy variables (Tanzania as reference) demonstrated relatively good fit (CFI = 0.922, RMSEA = 0.045, SRMR = 0.051). Based on modification indices from the multi-group analyses, direct paths were included for three items identified as potential sources of non-invariance. Significant DIF emerged for all three: item 31 (“Do not understand other people’s feelings”) showed negative DIF in all countries relative to Tanzania, with particularly pronounced effects in Haiti (estimate = -1.50, *p* < .001). Item 32 (“Tease others”) showed positive DIF in Ghana (estimate = + 0.81, *p* < .001) and Uganda (estimate = + 0.21, *p* < .001), while item 27 (“Have less fun with doing things”) showed negative DIF in both Ghana (estimate = -0.65, *p* < .001) and Uganda (estimate = -0.46, *p* < .001). These findings confirm that the metric non-invariance observed in the multi-group analysis is driven by systematic cross-country differences in these specific items.

MIMIC models estimated separately for each country with gender as a covariate demonstrated comparably strong fit in Tanzania (CFI = 0.950), Ghana (CFI = 0.967), Uganda (CFI = 0.962), and Pakistan (CFI = 0.952), while model fit in Haiti was considerably worse (CFI = 0.897).

MIMIC models estimated for Tanzania, Ghana, and Uganda with school level (secondary versus primary) as a covariate could be considered to fit well. In Tanzania (CFI = 0.938), significant negative effects of secondary school emerged on all three factors, indicating lower factor means among secondary students for Internalizing (estimate = -0.12, *p* < .001), Externalizing (estimate = -0.28, *p* < .001), and Attention (estimate = -0.23, *p* < .001). Uganda (CFI = 0.958) showed a more complex pattern: positive effects on Internalizing (estimate = + 0.12, *p* < .001) and Attention (estimate = + 0.08, *p* < .05), alongside a negative effect on Externalizing (estimate = -0.10, *p* < .01). For Ghana (CFI = 0.965), direct paths from school level to the five items freed in the partial invariance model revealed significant DIF for three items. Items 31 (“Do not understand other people’s feelings”) (estimate = -0.59, *p* < .001) and 16 (“Fight with other children”) (estimate = -0.36, *p* < .001) showed negative DIF, indicating higher endorsement among primary students, while item 10 (“Afraid of new situations”) showed positive DIF (estimate = + 0.22, *p* < .01), reflecting higher endorsement among secondary students. Items 21 (“Have trouble sleeping”) and 30 (“Do not show feelings”) did not show significant DIF (*p* > .05), suggesting these items function equivalently across age groups despite being freed in the multi-group analysis.

## Discussion

Establishing measurement invariance of self-report instruments for child and adolescent mental health is essential for making valid cross-group comparisons, thereby advancing equitable global mental health research and practice. The present study provides one of the most comprehensive evaluations to date of the measurement invariance of the 35-item Pediatric Symptom Checklist youth self-report (PSC-Y) across five Global South countries, offering critical insights into how this screening tool functions across diverse cultural, developmental, and gender-based groups.

### Cross-cultural structure and invariance

A central finding is the consistent emergence of a strong general factor underlying children’s responses across all five countries. Convergent evidence from the bifactor ESEM models, with omega hierarchical values exceeding 0.90 in every sample; the second-order models, with the three first-order factors loading strongly on the higher-order factor; and the three-factor correlated models, with high inter-factor correlations, indicates that the majority of reliable item-level variance is attributable to a common dimension of psychological distress. This consistency across modeling approaches underscores the robustness of the general factor and provides support for a latent p factor of psychopathology in childhood and adolescence [74, 75], a concept primarily developed in Western samples. Given that previous studies of the PSC in non-Western settings, including Zambia [42] and Botswana [41], similarly found a strong general factor, whereas studies in Western contexts observed more differentiated three-factor structures [46–48], our findings suggest that responses to the PSC-Y in non-Western settings may reflect a more integrated pattern of psychological distress than the discrete diagnostic categories developed in Western contexts would imply. At the same time, the three specific problem factors (internalizing, externalizing, attention) showed meaningful differentiation that was largely consistent across countries. Because they are highly correlated with the general factor and with each other, subscale scores are heavily influenced by overall distress and may not reflect distinct, separable dimensions of psychopathology in these settings. Rather, they may offer clinically useful information about the predominant domain of difficulty; for example, whether a child’s distress manifests primarily through emotional, behavioral, or attentional symptoms.

However, the strong general factor does not mean that the total score measures the same construct equivalently across countries. While configural invariance was supported across all five countries, indicating that the same underlying constructs are measured everywhere, metric invariance was violated. This was also the case in a post-hoc analysis only including the three African countries, suggesting that measurement non-invariance is not solely a function of broad cultural differences between continents, but can emerge even among culturally more similar countries. This indicates that, although the items may share substantial common variance within each setting, the way each item relates to the underlying construct differs systematically across settings. Thus, a child with a given total score in Tanzania and a child with the same total score in Haiti may not have the same underlying level of psychological distress, because the items contributing to those scores function differently in the two contexts. This failure to achieve metric invariance is consistent with studies on the cross-cultural invariance of the SDQ [26, 31], highlighting the general challenges in achieving measurement equivalence across diverse cultural contexts. These challenges arise in part because psychological symptoms may be understood, experienced, and expressed differently across cultures [10, 11].

In line with this, we observed a systematic pattern of non-invariance, with items related to empathy (item 31: “I do not understand what other people are feeling”), peer interactions (item 32: “I like to tease and make fun of others”) and diminished enjoyment (item 27: “I have less fun with doing things than I used to.”) showing the most consistent differential functioning across countries. Importantly, measurement non-invariance need not be viewed solely as a statistical nuisance; it can also reveal meaningful cultural variation in how psychological constructs are understood [76, 77]. From this perspective, the differential functioning observed for these items suggests that the meaning of these constructs may differ across contexts rather than simply indicating item “bias” [78].

Crucially, norms around emotional expression and interpersonal harmony vary substantially across cultures. What constitutes ‘understanding’ another person’s feelings may be interpreted differently across, and even within, cultural contexts, reflecting variation in models of self and social relationships, as well as display rules regarding emotional expression [79, 80]. In some cultural contexts, emotional understanding is communicated through non-verbal cues, shared social knowledge, and community-oriented frameworks rather than through explicit emotional labeling [81, 82]. Socialization practices around peer interactions also vary considerably across cultural contexts. In some settings, teasing among peers may be perceived as normative and even viewed as a form of social bonding or playful interaction rather than a sign of externalizing problems [18]. Furthermore, differences in cultural norms around play, leisure, and emotional expression may influence the interpretation and reporting of the item on diminished enjoyment of activities.

These findings also raise important questions about the cultural assumptions embedded in the PSC-Y. Originally developed and tested in Western contexts, the instrument reflects diagnostic frameworks that may not fully align with local understandings of distress. When measurement properties vary systematically across populations, this has implications for the fairness and equity of using the instrument for cross-cultural comparisons. At the same time, other factors may also contribute to the observed differences, including substantial heterogeneity even within countries, the specific communities sampled, and nuances in translation that could affect how items are interpreted.

### Gender and school-level invariance

We observed full measurement invariance across gender in all five countries, suggesting that the PSC-Y functions equivalently for boys and girls in the settings studied. MIMIC analyses provided convergent validation, with models including gender as a covariate demonstrating strong fit in four out of five countries. In Haiti, model fit was somewhat weaker; thus, the gender invariance findings for this sample should be interpreted with greater caution. From an equity perspective, these findings suggest that in most settings, the instrument does not systematically over-pathologize or under-identify mental health difficulties in either gender.

Invariance across school level (as a proxy for developmental stage) showed a more complex pattern across the three African samples in which both age groups were represented. Tanzania demonstrated full invariance, Uganda met conventional cut-offs, and Ghana required partial invariance after freeing five items, particularly those related to empathy (item 31), fighting (item 16), and anxiety (item 10). MIMIC analyses corroborated these patterns, confirming that the former two items were the primary sources of age-related differential functioning in the Ghanaian sample.

This pattern likely reflects culturally specific developmental expectations. However, it is important to note that these findings represent school-level differences which may be due to educational experiences, social context, or other factors beyond developmental maturation. Younger children’s higher endorsement of fighting items may reflect normative conflict resolution processes in middle childhood, consistent with predominantly Western studies showing that physical expressions of conflict tend to decline with age [83, 84]. As children mature into adolescence, expectations around emotional regulation and appropriate conflict resolution shift, potentially leading older children to interpret and endorse such items differently [85]. Similarly, empathy-related items may function differently across age groups because the ability to understand others’ feelings may be seen as a developmental milestone that is increasingly expected with age in the Ghanaian context, such that older adolescents who endorse difficulties with empathy may signal more clinically meaningful difficulties than younger children for whom such understanding is still developing [85]. Item 10 (“I am afraid of new situations”) showed higher endorsement among older students, which may reflect the developmental transition to adolescence, where anxiety around novel social situations, particularly in the context of changing school environments, becomes more salient [86].

The absence of age-related DIF for these items in Tanzania and Uganda suggests that developmental transitions in those settings may be more culturally continuous, or that the items tap aspects of experience that are less age-graded. Together, these findings underscore the importance of verifying invariance before making age comparisons and highlight the need for developmental and cultural contextualization when interpreting age differences in psychological distress.

### Limitations

Several limitations guide interpretation of the present findings. First, while the sample was large and geographically diverse, it was drawn from specific communities and school systems within each country and the results may not generalize to other regions, populations, or linguistic groups.

Second, the study relied exclusively on youth self-report collected via structured interviews rather than self-administered questionnaires. This limits comparability to typical screening practice where children complete the instrument independently, and may have influenced responses through social desirability or interviewer effects.

Third, although the PSC-Y has been validated for youth ages 9 and above [55], our sample included children as young as 6 years. While the structured interview format with trained assessors may have facilitated comprehension in younger participants, the appropriateness of the instrument for this age group cannot be assured, and findings involving the youngest children should be interpreted cautiously.

Fourth, the cross-sectional design precludes examination of developmental trajectories and stability of the factor structure over time. Consequently, we cannot determine whether the strong general factor reflects a stable trait-like dimension or varies with age and life experiences.

Fifth, age groups were defined using school level as a proxy for developmental stage. While this approach is common in school-based research and was feasible in Tanzania, Ghana, and Uganda, school level is an imperfect proxy for chronological age, and age is an imperfect proxy for cognitive and emotional maturity. School systems vary significantly across the three African countries, and the meaning of ‘primary’ versus ‘secondary’ schooling differs across contexts. Additionally, we could not examine age-related invariance in Pakistan or Haiti, where samples comprised a single school level.

Sixth, gender was assessed using a binary measure. This omits the full diversity of gender identity and limits the applicability of our findings to non-binary and gender-diverse youth, for whom instrument functioning may differ.

Seventh, while translation quality was considered, linguistic nuances may still influence findings. The differential functioning observed for several items could reflect translation challenges rather than genuine cultural differences in symptom meaning, which cannot be disentangled with quantitative methods alone. The adaptation of item 19 from “I am down on myself” to “I think badly of myself” may be a case in point; while this revision was made to enhance clarity and developmental appropriateness, it may not fully capture the intended construct across languages and cultural contexts.

Eighth, the study did not examine predictive validity, sensitivity to change, or clinical utility, and the established cutoff of 28 has not been validated in these contexts. This means that while we have established structural validity and invariance, we cannot speak to whether the PSC-Y accurately identifies clinically significant distress or detects changes following intervention in these settings.

Ninth, school-based samples may not represent out-of-school children. Given that out-of-school children may experience different patterns of psychological distress and face greater adversities, the instrument’s performance in this vulnerable population remains unknown.

Tenth, our analyses did not include socioeconomic status, which may influence stress exposure, educational opportunities, perceptions of mental health, symptom reporting, and access to resources [87]. Without this information, we cannot determine whether observed differences in PSC-Y functioning across countries reflect cultural factors, socioeconomic conditions, or their interaction, limiting the conclusions we can draw about the sources of cross-country variation in measurement properties.

Eleventh, the use of different models for within-country structural analyses (bifactor ESEM), cross-country invariance testing (second-order CFA), and subgroup analyses (three-factor correlated model) represents a methodological limitation. While the ideal approach would have been to use the same model across all analytical stages, these models converge on the same substantive conclusion, that a strong general factor accounts for the majority of reliable variance.

Finally, broader epistemological questions remain about whether a Western-derived instrument can fully capture local understandings of distress. The pursuit of measurement invariance, while methodologically rigorous, risks imposing external categories on local experience, and the strong general factor observed may reflect shared method variance rather than genuinely integrated experiences of distress across all cultural contexts.

### Implications for research and practice

The present findings provide clear guidance for research and practice, with the important caveat that invariance must be re-evaluated in new contexts. Because metric invariance was not established across countries, our data do not support cross-country comparisons of PSC-Y scores. This means that, at present, the instrument cannot be considered truly equitable for cross-cultural applications, for example to compare prevalence rates, allocate resources, or evaluate intervention effects across countries. Investigators should not use total scores or subscale scores to compare distress levels across the five countries examined in this study, nor should they pool scores across countries without accounting for systematic measurement differences, for example, by using multi-group models that allow parameters to differ across groups. For within-country research, full gender invariance justifies direct comparisons of boys and girls on all scores, including the use of gender as a covariate and the testing of gender-by-intervention interactions without concern for measurement bias. Age comparisons appear to be valid in Tanzanian and Ugandan samples, while Ghanaian samples require partial invariance models or exclusive use of total scores.

For clinical practice, the strong general factor indicates that total scores can serve as pragmatically useful indicators for screening purposes within each country. However, the finding that the PSC-Y functions differently across cultural contexts raises important questions about the appropriateness of a single universal cutoff score. While the commonly used cutoff of 28 may be appropriate in some settings [44], our results suggest that optimal thresholds may vary across cultural groups. Clinicians should therefore exercise caution when applying this cutoff in contexts where it has not been locally validated and should supplement it with clinical judgment and local knowledge. Subscale scores can offer clinically useful information about predominant domains of difficulty but should be interpreted alongside total scores, as they are substantially influenced by the general factor. Gender differences can be interpreted as genuine, while age comparisons require context-specific caution.

### Future directions

Future work should prioritize qualitative methods such as cognitive interviewing and focus groups to understand how children interpret PSC-Y items across cultural contexts, whether differential functioning reflects genuine differences in symptom meaning, and whether the Western conceptual framework embedded in the instrument aligns with local understandings of distress. Ideally, such qualitative work would precede or accompany quantitative validation to ensure instruments are culturally grounded from the outset [11, 88]. However, given the urgent need for screening tools in many Global South settings and the resource constraints that limit ground-up instrument development, rigorous quantitative evaluation of available instruments represents a necessary step in the interim. The present study provides this quantitative foundation, but it is not a substitute for the deeper qualitative work that should follow. As our findings illustrate, ongoing local validation is critical in all settings.

In addition to these qualitative priorities, future research should also establish locally appropriate cutoff scores through validation against gold-standard assessments, examining whether optimal thresholds vary across cultural contexts for both total and subscale scores. Studies should extend cross-country comparisons to additional Global South settings to examine whether the patterns observed in the present study generalize more broadly or reveal new forms of cultural variation. Such studies should continue to prioritize sampling across diverse regions, linguistic groups, and socioeconomic strata, and additionally include vulnerable groups such as out-of-school children who may experience different patterns of psychological distress. Furthermore, studies should employ longitudinal designs to examine stability of the factor structure over time and across developmental transitions. In addition, incorporating multi-informant (parent, teacher) reports is important to clarify whether psychometric properties generalize across informants.

Future invariance research should consider emerging methodological advancements such as dynamic measurement invariance (DMI) cutoffs [89]. Traditional invariance testing relies on fixed cut-offs (e.g., ΔCFI ≤ 0.01), but recent work demonstrates that these thresholds may not generalize well across different model and data characteristics. DMI addresses this limitation by executing simulations based on the researcher’s specific model and data to derive custom fit index difference cut-offs with optimal performance for the model being evaluated [89]. Applying such approaches in future cross-cultural studies could provide more accurate and context-sensitive evaluations of measurement invariance. In addition, future research must examine predictive validity, sensitivity to change, and utility for treatment monitoring to strengthen the evidence base for using the PSC-Y in intervention research and clinical practice. While the present study provides a ​methodological template, continued, contextually grounded research using invariance testing as routine practice is required for the PSC-Y to realize its potential as a tool for equitable global child mental health screening.

## Conclusions

This study provides robust evidence that the PSC-Y demonstrates strong structural validity for assessing child psychological distress within each of the five Global South samples examined. The consistent strong general factor supports its use as a total screening score for identifying children at elevated risk within each country. However, because metric invariance was not established across countries, the present findings do not support cross-country comparisons of PSC-Y total or subscale scores. Full gender invariance across all five samples is an exceptional finding that validates direct comparisons between boys and girls within countries, while age-related findings highlight the importance of developmental context, with Tanzania and Uganda showing strong invariance and Ghana requiring partial invariance. For now, we recommend using the PSC-Y as a country-specific measure for comparative purposes and avoiding cross-national inferences based on observed scores. Within countries, the instrument’s strong general factor and gender invariance make it a valuable tool for screening, while subscale scores may offer clinically useful descriptive information about predominant domains of difficulty but should be interpreted with caution given their substantial overlap with the general factor. These psychometric strengths, combined with the instrument’s practical advantages of being open-access, relatively brief, and free of reverse-worded items, position the PSC-Y as a valuable tool for child and adolescent mental health screening within diverse Global South settings, provided that it is used within its demonstrated limits and accompanied by ongoing cultural adaptation and local validation.

## Supplementary Information

Below is the link to the electronic supplementary material.

Supplementary Information

## Data Availability

The data analyzed in this study are derived from three cluster-randomized controlled trials. Due to the sensitive nature of the data and ethicalrestrictions charpf F, Mattonet K, Hecker T. Factor structure and measurement invariance of the 35-item self-report version of the PediatricSymptom Checklist (PSC-35) in children and adolescents in five Global South Countries. OSF. 2025. Available from: https://osf.io/tvpimposedby the institutional review boards of the participating countries, the raw data cannot be made publicly available. The complete R syntax for allmodels and preregistered analysis plan are available on the Open Science Framework (OSF) at https://osf.io/tvp8d/overview [90], enabling verification of the analytical procedures. The Pediatric Symptom Checklist – Youth Report (PSC-Y) is an open-access instrument developed by Jellinek and colleagues [36]. The English version of the 35-item PSC-Y is available at https://www.brightfutures.org/mentalhealth/pdf/professionals/ped_sympton_chklst.pdf , and translated versions used in this study are availableon the OSF repository at https://osf.io/tvp8d/overview [90]. Requests for data access may be considered by the corresponding author inaccordance with the data sharing agreements of the original trials. 36. Jellinek MS, Murphy JM, Little M, Pagano ME, Comer DM, Kelleher KJ. Use of the Pediatric Symptom Checklist to screen for psychosocial problems in pediatric primary care: A national feasibility study. Arch Pediatr Adolesc Med. 1999;153(3):254–60. 90. Scharpf F, Mattonet K, Hecker T. Factor structure and measurement invariance of the 35-itemself-report version of the Pediatric Symptom Checklist (PSC-35) in children and adolescents in five Global South Countries. OSF. 2025.Available from: https://osf.io/tvp8d/.

## Abbreviations

CFA: Confirmatory Factor Analysis
CFI: Comparative Fit Index
DIF: Differential Item Functioning
DMI: Dynamic Measurement Invariance
ESEM: Exploratory Structural Equation Modeling
LMICs: Low- and Middle-Income Countries
MIMIC: Multiple Indicators Multiple Causes
OSF: Open Science Framework
PSC: Pediatric Symptom Checklist
PSC-17: 17-item Pediatric Symptom Checklist
PSC-35: 35-item Pediatric Symptom Checklist
PSC-Y: Pediatric Symptom Checklist – Youth Report
RMSEA: Root Mean Square Error of Approximation
SDQ: Strengths and Difficulties Questionnaire
SRMR: Standardized Root Mean Square Residual
WLSMV: Weighted Least Square Mean and Variance Adjusted Estimator
YSR: Youth Self-Report
