# Supplementary material for: Cross-cultural validity of the Pediatric Symptom Checklist – Youth Report (PSC-Y) in five ​Global ​South countries: Implications for equitable child mental health screening
Source: BMC Med. 2026 Nov 4;24:620. doi: 10.1186/s12916-026-05383-w (PMC13744113; doi:10.1186/s12916-026-05383-w)
Supplement: Supplementary file 1 [file 12916_2026_5383_MOESM1_ESM.docx]

**Additional file 1: Table S1**
*Languages used for PSC-Y Administration by Country*

| **Country** | **Primary Language(s)** | **Notes** |
| --- | --- | --- |
| Tanzania | Kiswahili | National language |
| Ghana | English, Fante (Mfantseman District), Twi (Techiman District), Moar (Bunkpurugu District) | Local languages used where needed |
| Uganda | English, Luganda (Kayunga District), Lhukonzo (Kasese District), Acholi (Pader District) | Local languages used where needed |
| Haiti | Kreyol | Haitian Creole (Kreyòl) |
| Pakistan | Urdu | National language |

*Note*. Complete translated versions of the PSC-Y for each language are available on the Open Science Framework (OSF):

**Additional file 2: Table S2**
*Pediatric Symptom Checklist - Youth Self-Report (PSC-Y): Item Wording and Factor Assignment*

| **Item No.** | **Item Wording** | **Dimension** |
| --- | --- | --- |
| PSC_1 | I have aches and pains in my head, stomach or other body parts. | Internalizing |
| PSC_2 | I prefer to spend time alone rather than with others. | Internalizing |
| PSC_3 | I get tired easily, I have little energy. | Internalizing |
| PSC_4 | I am fidgeting and cannot sit still for long. | Attention |
| PSC_5 | I have trouble with the teachers. | Externalizing |
| PSC_6 | I am not interested in what I learn at school. | Attention |
| PSC_7 | I feel like I always have to move around and do something. | Attention |
| PSC_8 | I daydream too much and have my mind somewhere else. | Attention |
| PSC_9 | I get distracted easily. | Attention |
| PSC_10 | I am afraid of new situations. | Internalizing |
| PSC_11 | I feel sad and unhappy. | Internalizing |
| PSC_12 | I get angry easily. | Externalizing |
| PSC_13 | I feel hopeless. | Internalizing |
| PSC_14 | I have difficulties concentrating on the things I do. | Attention |
| PSC_15 | I am not very interested in being with my friends. | Internalizing |
| PSC_16 | I fight with other children. | Externalizing |
| PSC_17 | I am often absent from school. | Externalizing |
| PSC_18 | I am not doing well in school. | Externalizing |
| PSC_19 | I think badly of myself. | Internalizing |
| PSC_20 | I believe I may be seriously ill. | Internalizing |
| PSC_21 | I have difficulties with sleeping. | Internalizing |
| PSC_22 | I worry a lot. | Internalizing |
| PSC_23 | I want to be with my parents more than I used to. | Internalizing |
| PSC_24 | I feel that I am a bad person. | Internalizing |
| PSC_25 | I like to do things that are dangerous or risky. | Externalizing |
| PSC_26 | I get hurt frequently. | Externalizing |
| PSC_27 | I have less fun with doing things than I used to. | Internalizing |
| PSC_28 | I do things that I am too old for. | Attention |
| PSC_29 | I do not listen to rules. | Externalizing |
| PSC_30 | I do not show my feelings. | Internalizing |
|  |  |  |
| **Item No.** | **Item Wording** | **Dimension** |
| PSC_31 | I do not understand what other people are feeling. | Externalizing |
| PSC_32 | I like to tease and make fun of others. | Externalizing |
| PSC_33 | I blame others for problems that I have. | Externalizing |
| PSC_34 | I take things that are not mine. | Externalizing |
| PSC_35 | I do not want to share with others. | Externalizing |

**Additional file 3: Table S3a**

*Descriptive Statistics, Floor and Ceiling Effects, Skewness, and Kurtosis for the PSC Items Across Countries (Tanzania, Ghana, Uganda)*

|  | Tanzania |  |  |  |  | Ghana |  |  |  |  | Uganda |  |  |  |  |
| --- | --- | --- | --- | --- | --- | --- | --- | --- | --- | --- | --- | --- | --- | --- | --- |
|  | *M (SD)* | Floor | Ceiling | Skew | Kurtosis | *M (SD)* | Floor | Ceiling | Skew | Kurtosis | *M (SD)* | Floor | Ceiling | Skew | Kurtosis |
| PSC_1 | 0.96 (0.77) | 0.32 | 0.28 | 0.07 | -1.31 | 0.87 (0.66) | 0.29 | 0.16 | 0.15 | -0.76 | 0.90 (0.63) | 0.26 | 0.16 | 0.09 | -0.55 |
| PSC_2 | 0.34 (0.66) | 0.77 | 0.11 | 1.73 | 1.46 | 0.44 (0.68) | 0.67 | 0.11 | 1.25 | 0.22 | 0.44 (0.69) | 0.67 | 0.11 | 1.24 | 0.17 |
| PSC_3 | 0.49 (0.72) | 0.65 | 0.13 | 1.12 | -0.20 | 0.47 (0.63) | 0.60 | 0.07 | 0.97 | -0.13 | 0.61 (0.69) | 0.50 | 0.12 | 0.67 | -0.70 |
| PSC_4 | 0.52 (0.68) | 0.58 | 0.11 | 0.93 | -0.36 | 0.46 (0.65) | 0.63 | 0.09 | 1.09 | 0.00 | 0.38 (0.63) | 0.70 | 0.08 | 1.43 | 0.83 |
| PSC_5 | 0.20 (0.52) | 0.85 | 0.05 | 2.53 | 5.28 | 0.05 (0.24) | 0.95 | 0.00 | 4.76 | 23.99 | 0.19 (0.44) | 0.83 | 0.02 | 2.20 | 4.19 |
| PSC_6 | 0.18 (0.51) | 0.88 | 0.06 | 2.78 | 6.54 | 0.23 (0.52) | 0.82 | 0.05 | 2.25 | 4.07 | 0.23 (0.52) | 0.81 | 0.04 | 2.16 | 3.76 |
| PSC_7 | 0.41 (0.64) | 0.68 | 0.09 | 1.30 | 0.47 | 0.54 (0.64) | 0.54 | 0.08 | 0.78 | -0.45 | 0.67 (0.69) | 0.46 | 0.13 | 0.54 | -0.80 |
| PSC_8 | 0.38 (0.64) | 0.70 | 0.09 | 1.43 | 0.78 | 0.43 (0.60) | 0.63 | 0.06 | 1.05 | 0.09 | 0.46 (0.61) | 0.60 | 0.06 | 0.96 | -0.11 |
| PSC_9 | 0.57 (0.71) | 0.56 | 0.13 | 0.83 | -0.61 | 0.44 (0.61) | 0.62 | 0.06 | 1.02 | 0.02 | 0.51 (0.59) | 0.55 | 0.05 | 0.71 | -0.47 |
| PSC_10 | 0.61 (0.72) | 0.53 | 0.14 | 0.73 | -0.75 | 0.60 (0.67) | 0.50 | 0.11 | 0.67 | -0.65 | 0.69 (0.67) | 0.43 | 0.12 | 0.47 | -0.79 |
| PSC_11 | 0.38 (0.65) | 0.72 | 0.10 | 1.49 | 0.86 | 0.46 (0.62) | 0.61 | 0.07 | 1.00 | -0.05 | 0.61 (0.63) | 0.47 | 0.08 | 0.54 | -0.64 |
| PSC_12 | 0.43 (0.69) | 0.68 | 0.12 | 1.29 | 0.25 | 0.44 (0.62) | 0.63 | 0.07 | 1.08 | 0.09 | 0.54 (0.67) | 0.57 | 0.10 | 0.88 | -0.41 |
| PSC_13 | 0.26 (0.55) | 0.79 | 0.05 | 1.98 | 2.90 | 0.17 (0.42) | 0.85 | 0.02 | 2.49 | 5.68 | 0.39 (0.58) | 0.67 | 0.05 | 1.22 | 0.49 |
| PSC_14 | 0.40 (0.62) | 0.67 | 0.07 | 1.28 | 0.50 | 0.39 (0.55) | 0.65 | 0.03 | 1.03 | 0.04 | 0.49 (0.60) | 0.56 | 0.05 | 0.78 | -0.38 |
| PSC_15 | 0.24 (0.57) | 0.83 | 0.07 | 2.27 | 3.82 | 0.24 (0.49) | 0.79 | 0.03 | 1.98 | 3.14 | 0.26 (0.54) | 0.79 | 0.05 | 1.98 | 2.93 |
| PSC_16 | 0.15 (0.39) | 0.87 | 0.02 | 2.70 | 6.89 | 0.48 (0.60) | 0.58 | 0.06 | 0.85 | -0.27 | 0.18 (0.41) | 0.84 | 0.01 | 2.20 | 4.11 |
| PSC_17 | 0.11 (0.37) | 0.91 | 0.02 | 3.63 | 13.00 | 0.28 (0.50) | 0.74 | 0.02 | 1.51 | 1.34 | 0.34 (0.53) | 0.68 | 0.03 | 1.19 | 0.37 |
| PSC_18 | 0.44 (0.61) | 0.63 | 0.06 | 1.07 | 0.09 | 0.49 (0.60) | 0.56 | 0.05 | 0.78 | -0.37 | 0.50 (0.63) | 0.58 | 0.07 | 0.88 | -0.27 |
| PSC_19 | 0.11 (0.37) | 0.91 | 0.02 | 3.45 | 11.87 | 0.10 (0.33) | 0.91 | 0.01 | 3.24 | 10.49 | 0.23 (0.47) | 0.80 | 0.02 | 1.93 | 2.95 |
| PSC_20 | 0.23 (0.52) | 0.81 | 0.04 | 2.14 | 3.67 | 0.20 (0.44) | 0.82 | 0.02 | 2.10 | 3.71 | 0.42 (0.59) | 0.64 | 0.05 | 1.09 | 0.18 |
| PSC_21 | 0.25 (0.56) | 0.81 | 0.06 | 2.14 | 3.37 | 0.18 (0.44) | 0.85 | 0.02 | 2.49 | 5.63 | 0.36 (0.59) | 0.70 | 0.06 | 1.43 | 1.00 |
| PSC_22 | 0.27 (0.54) | 0.78 | 0.05 | 1.90 | 2.65 | 0.30 (0.54) | 0.74 | 0.04 | 1.59 | 1.58 | 0.46 (0.59) | 0.59 | 0.05 | 0.88 | -0.22 |
| PSC_23 | 1.44 (0.78) | 0.18 | 0.62 | -0.94 | -0.71 | 0.88 (0.81) | 0.39 | 0.27 | 0.22 | -1.43 | 1.38 (0.76) | 0.17 | 0.56 | -0.77 | -0.87 |
| PSC_24 | 0.10 (0.38) | 0.92 | 0.03 | 3.87 | 14.66 | 0.10 (0.33) | 0.91 | 0.01 | 3.29 | 10.81 | 0.17 (0.40) | 0.84 | 0.01 | 2.24 | 4.21 |
| PSC_25 | 0.10 (0.36) | 0.91 | 0.02 | 3.63 | 13.25 | 0.30 (0.56) | 0.75 | 0.05 | 1.68 | 1.84 | 0.13 (0.38) | 0.89 | 0.02 | 3.02 | 8.96 |
| PSC_26 | 0.28 (0.58) | 0.78 | 0.06 | 1.90 | 2.45 | 0.32 (0.54) | 0.72 | 0.04 | 1.46 | 1.19 | 0.36 (0.54) | 0.67 | 0.03 | 1.16 | 0.33 |
| PSC_27 | 0.65 (0.81) | 0.57 | 0.22 | 0.73 | -1.10 | 0.29 (0.50) | 0.74 | 0.02 | 1.49 | 1.27 | 0.45 (0.66) | 0.64 | 0.09 | 1.15 | 0.09 |
| PSC_28 | 0.26 (0.52) | 0.78 | 0.04 | 1.85 | 2.56 | 0.26 (0.49) | 0.76 | 0.02 | 1.69 | 1.97 | 0.29 (0.53) | 0.75 | 0.04 | 1.69 | 1.95 |
| PSC_29 | 0.22 (0.53) | 0.83 | 0.05 | 2.35 | 4.45 | 0.35 (0.52) | 0.68 | 0.02 | 1.12 | 0.20 | 0.32 (0.59) | 0.75 | 0.07 | 1.69 | 1.70 |
| PSC_30 | 0.57 (0.76) | 0.60 | 0.17 | 0.91 | -0.71 | 0.50 (0.67) | 0.60 | 0.10 | 1.01 | -0.20 | 0.74 (0.73) | 0.44 | 0.17 | 0.46 | -1.04 |
| PSC_31 | 1.07 (0.86) | 0.34 | 0.41 | -0.13 | -1.64 | 0.57 (0.72) | 0.57 | 0.14 | 0.85 | -0.63 | 0.81 (0.74) | 0.39 | 0.20 | 0.31 | -1.12 |
| PSC_32 | 0.22 (0.50) | 0.81 | 0.04 | 2.18 | 3.94 | 0.64 (0.71) | 0.50 | 0.14 | 0.64 | -0.82 | 0.37 (0.60) | 0.69 | 0.06 | 1.38 | 0.83 |
| PSC_33 | 0.19 (0.47) | 0.85 | 0.04 | 2.53 | 5.66 | 0.19 (0.42) | 0.83 | 0.01 | 2.09 | 3.56 | 0.32 (0.52) | 0.71 | 0.03 | 1.38 | 0.95 |
| PSC_34 | 0.13 (0.38) | 0.89 | 0.02 | 3.08 | 9.32 | 0.11 (0.34) | 0.89 | 0.01 | 3.02 | 8.91 | 0.13 (0.36) | 0.87 | 0.01 | 2.59 | 5.99 |
| PSC_35 | 0.60 (0.80) | 0.60 | 0.20 | 0.84 | -0.91 | 0.41 (0.62) | 0.67 | 0.07 | 1.26 | 0.45 | 0.36 (0.61) | 0.71 | 0.07 | 1.49 | 1.05 |

**Additional file 3: Table S3b**

*Descriptive Statistics, Floor and Ceiling Effects, Skewness, and Kurtosis for the PSC Items Across Countries (Pakistan, Haiti)*

|  | Pakistan |  |  |  |  | Haiti |  |  |  |  |
| --- | --- | --- | --- | --- | --- | --- | --- | --- | --- | --- |
|  | *M (SD)* | Floor | Ceiling | Skew | Kurtosis | *M (SD)* | Floor | Ceiling | Skew | Kurtosis |
| PSC_1 | 0.77 (0.66) | 0.36 | 0.13 | 0.29 | -0.77 | 1.14 (0.66) | 0.16 | 0.30 | -0.17 | -0.76 |
| PSC_2 | 0.32 (0.65) | 0.78 | 0.10 | 1.78 | 1.67 | 0.44 (0.64) | 0.65 | 0.08 | 1.18 | 0.22 |
| PSC_3 | 0.70 (0.77) | 0.49 | 0.19 | 0.56 | -1.12 | 0.59 (0.68) | 0.52 | 0.11 | 0.74 | -0.62 |
| PSC_4 | 0.45 (0.66) | 0.64 | 0.09 | 1.15 | 0.10 | 0.76 (0.71) | 0.40 | 0.16 | 0.38 | -0.99 |
| PSC_5 | 0.12 (0.39) | 0.91 | 0.02 | 3.51 | 12.10 | 0.11 (0.38) | 0.91 | 0.02 | 3.54 | 12.39 |
| PSC_6 | 0.12 (0.40) | 0.91 | 0.03 | 3.61 | 12.58 | 0.11 (0.39) | 0.92 | 0.03 | 3.77 | 13.71 |
| PSC_7 | 0.86 (0.83) | 0.43 | 0.28 | 0.27 | -1.50 | 1.09 (0.69) | 0.20 | 0.28 | -0.11 | -0.88 |
| PSC_8 | 0.46 (0.68) | 0.64 | 0.11 | 1.15 | 0.02 | 0.68 (0.69) | 0.45 | 0.13 | 0.52 | -0.84 |
| PSC_9 | 0.52 (0.70) | 0.60 | 0.12 | 0.96 | -0.37 | 0.44 (0.64) | 0.63 | 0.08 | 1.12 | 0.13 |
| PSC_10 | 0.63 (0.74) | 0.53 | 0.16 | 0.72 | -0.86 | 0.63 (0.68) | 0.48 | 0.12 | 0.62 | -0.73 |
| PSC_11 | 0.39 (0.63) | 0.69 | 0.08 | 1.36 | 0.68 | 0.85 (0.70) | 0.33 | 0.18 | 0.22 | -0.96 |
| PSC_12 | 0.82 (0.83) | 0.45 | 0.27 | 0.35 | -1.46 | 0.69 (0.73) | 0.47 | 0.16 | 0.55 | -0.97 |
| PSC_13 | 0.24 (0.50) | 0.79 | 0.03 | 1.93 | 2.89 | 0.47 (0.68) | 0.63 | 0.10 | 1.10 | -0.06 |
| PSC_14 | 0.43 (0.65) | 0.66 | 0.09 | 1.22 | 0.27 | 0.66 (0.70) | 0.48 | 0.13 | 0.59 | -0.81 |
| PSC_15 | 0.16 (0.45) | 0.87 | 0.03 | 2.86 | 7.51 | 0.37 (0.62) | 0.71 | 0.08 | 1.45 | 0.92 |
| PSC_16 | 0.15 (0.41) | 0.88 | 0.02 | 2.90 | 8.07 | 0.44 (0.68) | 0.66 | 0.11 | 1.23 | 0.19 |
| PSC_17 | 0.18 (0.42) | 0.84 | 0.02 | 2.27 | 4.52 | 0.32 (0.56) | 0.73 | 0.05 | 1.58 | 1.51 |
| PSC_18 | 0.22 (0.46) | 0.81 | 0.02 | 2.04 | 3.44 | 0.44 (0.65) | 0.64 | 0.09 | 1.17 | 0.18 |
| PSC_19 | 0.15 (0.42) | 0.88 | 0.03 | 3.00 | 8.52 | 0.17 (0.44) | 0.85 | 0.03 | 2.59 | 6.19 |
| PSC_20 | 0.23 (0.51) | 0.81 | 0.04 | 2.16 | 3.80 | 0.56 (0.64) | 0.53 | 0.08 | 0.72 | -0.51 |
| PSC_21 | 0.21 (0.50) | 0.83 | 0.04 | 2.37 | 4.78 | 0.42 (0.65) | 0.67 | 0.09 | 1.30 | 0.43 |
| PSC_22 | 0.23 (0.49) | 0.80 | 0.03 | 2.07 | 3.48 | 0.47 (0.65) | 0.62 | 0.09 | 1.06 | -0.03 |
| PSC_23 | 1.58 (0.71) | 0.13 | 0.71 | -1.36 | 0.32 | 1.61 (0.59) | 0.06 | 0.67 | -1.26 | 0.54 |
| PSC_24 | 0.11 (0.35) | 0.91 | 0.02 | 3.53 | 12.59 | 0.08 (0.32) | 0.94 | 0.02 | 4.48 | 20.65 |
| PSC_25 | 0.17 (0.49) | 0.88 | 0.05 | 2.91 | 7.30 | 0.16 (0.45) | 0.87 | 0.03 | 2.83 | 7.39 |
| PSC_26 | 0.42 (0.64) | 0.67 | 0.09 | 1.27 | 0.39 | 0.54 (0.67) | 0.55 | 0.10 | 0.83 | -0.45 |
| PSC_27 | 0.32 (0.58) | 0.74 | 0.06 | 1.66 | 1.68 | 0.29 (0.56) | 0.76 | 0.05 | 1.79 | 2.18 |
| PSC_28 | 0.42 (0.66) | 0.67 | 0.10 | 1.28 | 0.36 | 0.19 (0.50) | 0.85 | 0.05 | 2.59 | 5.72 |
| PSC_29 | 0.18 (0.47) | 0.86 | 0.04 | 2.67 | 6.38 | 0.36 (0.58) | 0.69 | 0.05 | 1.33 | 0.77 |
| PSC_30 | 0.59 (0.77) | 0.59 | 0.18 | 0.86 | -0.80 | 0.45 (0.72) | 0.69 | 0.14 | 1.26 | 0.07 |
| PSC_31 | 0.53 (0.75) | 0.62 | 0.15 | 0.99 | -0.51 | 0.24 (0.51) | 0.80 | 0.04 | 2.03 | 3.28 |
| PSC_32 | 0.10 (0.36) | 0.92 | 0.02 | 3.83 | 14.70 | 0.49 (0.72) | 0.65 | 0.13 | 1.13 | -0.20 |
| PSC_33 | 0.08 (0.32) | 0.93 | 0.01 | 4.22 | 18.34 | 0.24 (0.51) | 0.80 | 0.04 | 2.03 | 3.31 |
| PSC_34 | 0.03 (0.19) | 0.97 | 0.00 | 6.99 | 53.56 | 0.10 (0.35) | 0.92 | 0.02 | 3.70 | 13.91 |
| PSC_35 | 0.38 (0.70) | 0.74 | 0.13 | 1.53 | 0.75 | 0.22 (0.54) | 0.83 | 0.06 | 2.34 | 4.35 |

**Additional file 4: Table S4**

*Standardized Loadings of First-Order Factors on the Higher-Order General Factor (Second-Order Model Without Cross-Loadings)*

| **Country** | **Internalizing** | **Externalizing** | **Attention** |
| --- | --- | --- | --- |
| Tanzania | .94 | .94 | .91 |
| Ghana | .86 | .89 | .93 |
| Uganda | .92 | .92 | .97 |
| Pakistan | .88 | .84 | .99 |
| Haiti | .98 | .81 | .93 |
| **Mean** | **.92** | **.88** | **.95** |

*Note*. All loadings are standardized and statistically significant (p < .001). Loadings represent the strength of the relationship between each first-order factor and the higher-order general factor. The overall mean loading across all factors and countries is .91.

**Additional file 5: Table S5**

*Factor Correlations from the Three-Factor Correlated Model*

| **Country** | **Internalizing ↔ Externalizing** | **Internalizing ↔ Attention** | **Externalizing ↔ Attention** |
| --- | --- | --- | --- |
| Tanzania | .88 | .86 | .86 |
| Ghana | .77 | .80 | .82 |
| Uganda | .84 | .89 | .89 |
| Pakistan | .74 | .87 | .84 |
| Haiti | .80 | .91 | .76 |
| **Mean** | **.81** | **.87** | **.83** |

*Note.* All correlations are standardized and statistically significant (p < .001).

**Additional file 6: Table S6**

*Reliability Estimates for Bifactor ESEM Models*

| Country | *ω* hierarchical (G) | *ω* total (INT) | *ω* total (EXT) | *ω* total (ATT) | ω hierarchical (INT) | ω hierarchical (EXT) | ω hierarchical (ATT) |
| --- | --- | --- | --- | --- | --- | --- | --- |
| **Tanzania** | .926 | .002 | .373 | .302 | .003 | .453 | .392 |
| **Ghana** | .927 | .626 | .443 | .125 | .699 | .508 | .237 |
| **Uganda** | .913 | .325 | .403 | .134 | .393 | .466 | .184 |
| **Pakistan** | .917 | .159 | .591 | .187 | .214 | .649 | .261 |
| **Haiti** | .907 | .074 | .494 | .247 | .100 | .551 | .315 |

*Note.* ω hierarchical (G) represents the proportion of variance in total scores attributable to the general factor. ω total represents the reliability of each subscale score, including variance from both the general and specific factors. ω hierarchical for specific factors represents the proportion of variance in subscale scores attributable to the specific factor after controlling for the general factor. Cronbach's alpha is not reported for ESEM models as the presence of cross-loadings violates the assumptions of essential tau-equivalence.

**Additional file 7: Table S7**

Measurement invariance across the three African countries (Tanzania, Ghana, Uganda)

| **Model** | **χ²** | **df** | **CFI** | **TLI** | **RMSEA** | **SRMR** | **ΔCFI** | **ΔRMSEA** |
| --- | --- | --- | --- | --- | --- | --- | --- | --- |
| Configural | 4861.8 | 1671 | .961 | .959 | .041 | .068 | — | — |
| Metric | 8078.1 | 1739 | .923 | .921 | .056 | .089 | −.038 | +.015 |
| Scalar | 5580.3 | 1739 | .953 | .952 | .044 | .070 | +.030 | −.012 |
| Strict | 8965.8 | 1809 | .913 | .914 | .058 | .089 | −.040 | +.015 |

**Note.** Metric invariance failed (ΔCFI = −.038, exceeding the −.01 cutoff), indicating that the PSC-Y does not measure the same construct with equivalent item-factor relationships across the three African countries. The most consistently problematic items were items 27 ("I have less fun with doing things than I used to"), 31 ("I do not understand what other people are feeling"), and 16 ("I fight with other children")—the same items identified as problematic in the full five-country comparison. Consequently, scalar and strict invariance models are not interpretable. The scalar model showed identical degrees of freedom and a negative chi-square difference relative to the metric model, reflecting model misspecification rather than evidence of invariance. The strict model produced warnings regarding negative latent variances. Only configural invariance is supported across the three African countries. This analysis was conducted post-hoc, at the item level, using baseline data.
